# Chondrosarcoma: biology, genetics, and epigenetics

**DOI:** 10.12688/f1000research.15953.1

**Published:** 2018-11-20

**Authors:** Warren A Chow

**Affiliations:** 1 Department of Medical Oncology & Therapeutics Research, City of Hope, 1500 E. Duarte Rd, Duarte, CA, 91010, USA

**Keywords:** chondrosarcoma, genetics, epigenetics biology

## Abstract

Chondrosarcomas constitute a heterogeneous group of primary bone cancers characterized by hyaline cartilaginous neoplastic tissue. They are the second most common primary bone malignancy. The vast majority of chondrosarcomas are conventional chondrosarcomas, and most conventional chondrosarcomas are low- to intermediate-grade tumors (grade 1 or 2) which have indolent clinical behavior and low metastatic potential. Recurrence augurs a poor prognosis, as conventional chondrosarcomas are both radiation and chemotherapy resistant. Recent discoveries in the biology, genetics, and epigenetics of conventional chondrosarcomas have significantly advanced our understanding of the pathobiology of these tumors and offer insight into potential therapeutic targets.

## Introduction

Chondrosarcoma (CS) is the collective term for a group of heterogeneous, generally slow-growing, primary malignant tumors of bone characterized by the formation of hyaline cartilaginous neoplastic tissue. They primarily affect adults and are the second most common primary solid tumor of bone after osteogenic sarcoma ^1^. CS account for approximately 3 new cases per 10 ^6^ population per year ^2^. The prognosis for the majority of patients with CS is favorable and correlates with histologic grade and adequate surgical margins ^3^. Development of pulmonary metastatic disease is typically an ominous sign, however, owing to chemotherapy and radiation resistance. This is a result of its underlying phenotype: poor vascularization, slow division rate, and hyaline cartilaginous matrix prohibiting access to the cells. Recent research on the biology, genetics, and epigenetics of CS will be reviewed.

More than 90% of CS are conventional CS ^3^. Approximately 90% of these are low- to intermediate-grade (grade 1–2) and behave indolently and rarely metastasize ^3^. Only 5–10% of conventional CS are grade 3 and have high metastatic potential ^4^. CS that arise unassociated with a pre-existing lesion are called primary CS, and they are known as secondary if they develop from a pre-existing benign cartilage tumor such as an enchondroma or osteochondroma. They are further classified as central when they arise within the medullary cavity and peripheral when they arise from the bone surface, generally from the cartilage cap of an exostosis. Primary CS are nearly always central; secondary CS can be central or peripheral ^5^. Although CS are considered both chemotherapy and radiation resistant, there are rare case reports of responses to conventional chemotherapeutic agents, including gemcitabine ^6, 7^.

Variant CS subtypes are significantly less common ^1, 4, 5^. Dedifferentiated CS develop when low-grade conventional CS transform into a high-grade sarcoma, most frequently exhibiting features of osteosarcoma, fibrosarcoma, or undifferentiated pleomorphic sarcoma (UPS). Mesenchymal CS (MCS) is a highly malignant tumor exhibiting a bimorphic histologic pattern, with a highly undifferentiated small round cell component admixed with islands of well-differentiated cartilage. The *HEY1–NCOA2* fusion has been identified in MCS ^8^. Delayed recurrences after more than 20 years are not uncommon ^9^. Clear cell CS is a low-grade malignant tumor that most commonly involves the epiphyses of the long bones; the tumor cells are clear because their cytoplasm contains large amounts of glycogen ^10^. Extraskeletal myxoid CS is also a slow-growing soft-tissue tumor containing prominent myxoid degeneration and characterized by a prolonged clinical course despite high rates of local recurrence and metastases ^11^. It is characterized by t(9;22)(q22;q12), fusing *EWSR1* to *NRA3* (genes formerly termed *CHN*, *TEC*, or *NOR1*) ^12^. Other translocation partners to *NRA3* include *TAF15* and *TCF12* ^13, 14^. Because of the rarity of these variant CS, the remainder of this review will be dedicated to the more-prevalent conventional CS subtype.

## Normal bone formation

**Figure 1.**
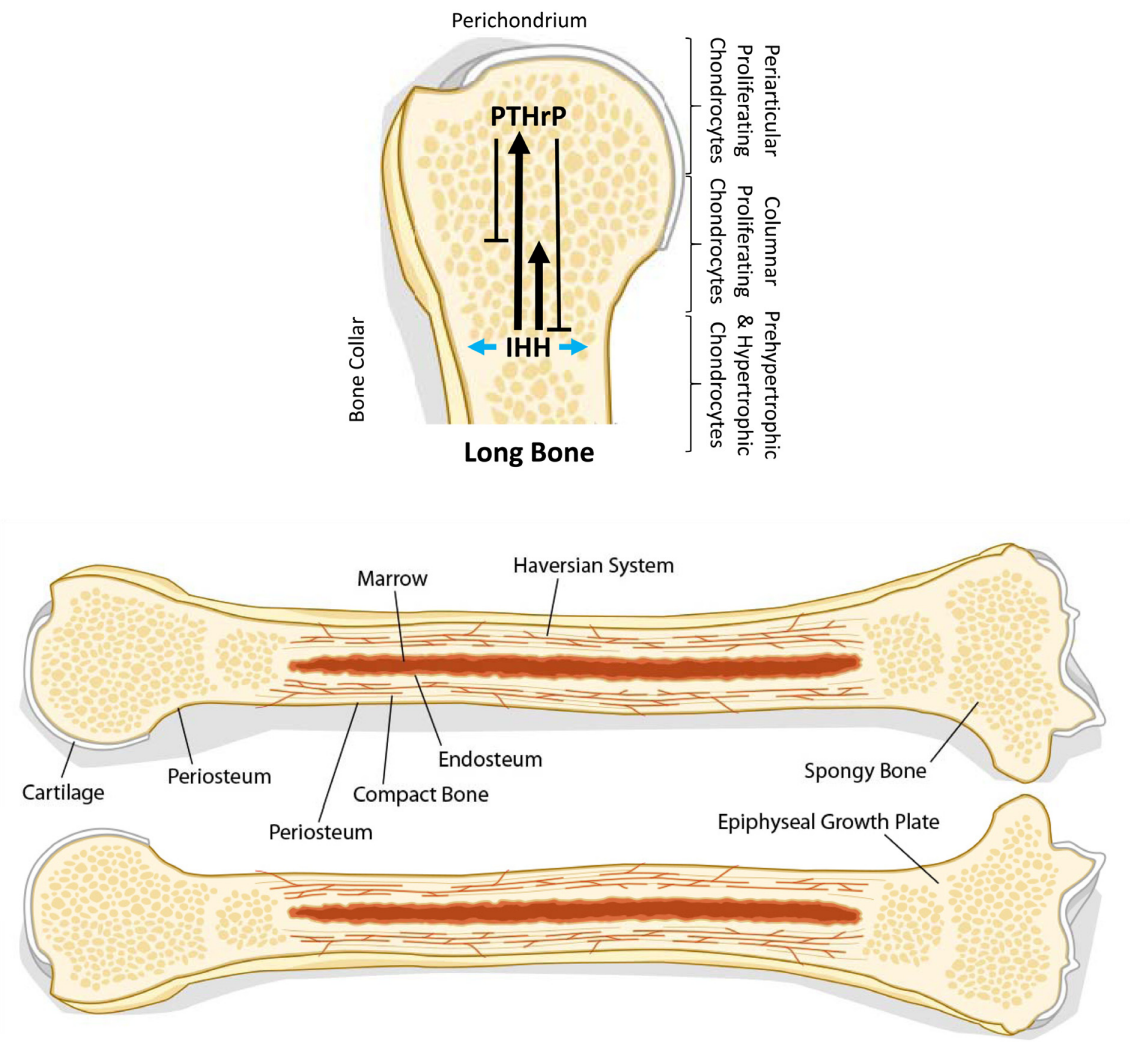
The IHH/PTHrP signaling pathway. Indian hedgehog (IHH) is secreted by prehypertrophic and hypertrophic chondrocytes. IHH stimulates the secretion of parathyroid hormone-related protein (PTHrP) in the periarticular growth plate by periarticular proliferating chondrocytes (long solid arrow). IHH also induces the division of columnar proliferating chondrocytes in a positive feedback loop (short solid arrow). PTHrP then acts on the columnar proliferating chondrocytes to inhibit their hypertrophic differentiation and maintain them in a proliferative state (short solid T-bar). PTHrP also inhibits IHH secretion via a negative feedback loop, eventually shutting down the proliferative signal (long solid T-bar). IHH also acts on perichondrial cells to induce mature osteoblasts, which form a bone collar (blue arrows). Adapted from Chung UI, Schipani E, McMahon AP, Kronenberg HM. **Indian hedgehog couples chondrogenesis to osteogenesis in endochondral bone development**. *J Clin Invest.* 2001; **107**:295-304.

Normal bone formation is a result of the production of hyaline cartilage matrix produced by chondrocytes, followed by ossification of the matrix by osteoblasts. First, mesenchymal stem cells differentiate at the epiphyseal growth plate into resting chondrocytes, which then migrate into a proliferative zone, where they multiply and increase the cellularity of the matrix ( Figure 1). These proliferative cells then leave the epiphyseal plate before undergoing a hypertrophic phase, whereby they mature into large clear cells. Eventually, the cells undergo apoptosis, leaving behind the characteristic hypocellular hyaline cartilage matrix. Osteoblasts then migrate into this matrix and initiate ossification ^15^.

## (Benign) cartilaginous neoplasms

Osteochondromas are benign cartilaginous neoplasms consisting of a cartilage-capped bony projection on the surface of the bone, containing a marrow cavity continuous with the underlying bone ^16^. They are one of the most common benign bone tumors. Approximately 15% of cases present with multiple lesions characteristic of the autosomal dominant multiple osteochondroma (MO) genetic disorder. The risk of malignant transformation to secondary peripheral CS is estimated at 1% for sporadic osteochondroma and less than 5% for MO ^17^. Biallelic inactivation of the exostosin glycosyltransferase ** ( *EXT1* or *EXT2*) genes is observed in the majority of both sporadic and hereditary osteochondroma cases ^18, 19^. The EXT proteins are required for the polymerization of heparan sulfate (HS) chains forming the hyaline cartilage.

Interestingly, the cells of peripheral CS are predominantly EXT1 positive ^20^, unlike the EXT1-negative cells within osteochondromas. Hence, EXT loss is likely an early event. The EXT-negative cells in osteochondroma create an extracellular mutation-promoting environment, whereupon neighboring EXT-positive cells acquire late-stage mutations that cause malignant transformation into CS.

Enchondromas are benign cartilaginous neoplasms that arise within the medullary cavity of bone, in contrast to osteochondromas, which arise on the surface. Histologically, they closely resemble grade 1 CS, and radiographic correlation for benign features is necessary for diagnosis. Mutations in the isocitrate dehydrogenase ( *IDH*) *1* and *2* genes are present in 85% of hereditary enchondromatosis-associated disorders, Ollier disease (enchondromatosis only), and Maffuci syndrome (enchondromatosis and hemangiomas) and 50% of solitary enchondromas ^21^. The risk of transformation into secondary central CS in patients affected by enchondromatosis is approximately 40% for Ollier disease and up to 53% in Maffuci syndrome ^22^.

*IDH* mutations are present in 52–59% of central CS and 57% of dedifferentiated CS ^23^. The presence of *IDH* mutations in benign enchondromas and malignant CS supports the notion that *IDH* mutations are an early event, and these cartilaginous neoplasms represent a spectrum of malignant potential. *IDH* mutations are found in gliomas, acute myeloid leukemia (AML), and cholangiocarcinomas ^24^. IDH is an enzyme involved in the tricarboxylic acid cycle (Krebs cycle), where it normally converts isocitrate to α-ketoglutarate (α-KG). Mutant *IDH* (m *IDH*) loses the ability to convert isocitrate to α-KG and gains a new function that leads to the accumulation of δ-2-hydroxyglutarate (D2HG). D2HG is considered an oncometabolite owing to its inhibition of α-KG-dependent dioxygenases involved in DNA and histone demethylation, which leads to a hypermethylated state of DNA and histones ^25^. However, clearly *IDH* mutations alone are insufficient for malignant transformation similar to EXT loss.

Inhibition of oncogenic m *IDH1/2* represents an opportunity for therapeutic intervention. Many of these inhibitors have been, or are being, evaluated in clinical trials for patients with AML or solid tumors, including CS ^26^. Agios Pharmaceuticals have the furthest clinically developed compounds. Ivosidenib (AG-120), an IDH1 inhibitor, and enasidenib (AG-221), an IDH2 inhibitor, are FDA approved in the U.S. for refractory AML with *mIDH1* and *mIDH2*, respectively. AG-881, a pan-mIDH1/2 inhibitor, is in early phase clinical testing. Likewise, IDH305 (Novartis), FT-2102 (Forma Therapeutics), and BAY1436032 (Bayer) are in early phase clinical testing. m *IDH* inhibition is a promising therapeutic approach in advanced CS.

## *COL2A1* 

The Cancer Genome Project (Wellcome Trust Sanger Institute, Cambridge, UK) performed the first large-scale whole-exome sequencing of 49 cases of 30 central, 4 peripheral, and 14 dedifferentiated CS along with matching normal tissue ^27^. In addition to confirming *IDH1/2* mutations in 59% of the cases, the investigators identified hypermutability of the major cartilage collagen gene *COL2A1*, with insertions, deletions, and rearrangements identified in 37% of cases. *COL2A1* encodes the α-chain of type II collagen fibers, the major collagen constituent of articular cartilage. Disruption of the collagen maturation process through production of aberrant pro-collagen α-chain is the likely result. The investigators hypothesized that *COL2A1* mutations might bring about fundamental perturbation of matrix deposition and signaling that contributes to oncogenesis through abrogation of normal differentiation programs in CS.

## Hedgehog pathway

The Hedgehog (HH) pathway is vital for normal embryogenesis and plays essential roles in the maintenance, renewal, and regeneration of adult tissue. The mammalian HH ligand Indian (IHH) initiates signaling through binding to the canonical membrane receptor Patched (PTCH1). IHH binding to PTCH1 results in derepression of Smoothened (SMO) and results in SMO accumulation. SMO mediates activation of the GLI transcription factor to initiate a series of cellular responses that range from survival and proliferation to cell fate specification and differentiation ^28^.

Chondrogenesis is regulated by the IHH/parathyroid hormone-related protein (PTHrP) pathway. IHH, the product of proliferating chondrocytes, self-induces chondrocyte cell division as well as the secretion of PTHrP by perichondrial chondrocytes. PTHrP inhibits chondrocyte differentiation and maintains them in a proliferative state. PTHrP also negatively regulates IHH in a negative feedback loop to allow the chondrocytes to differentiate in a controlled manner ( Figure 1).

Given the important role for HH signaling in chondrogenesis, therapeutic targeting of this pathway in CS has been investigated. The SMO inhibitors HPI-4 and IPI-926 have been reported to inhibit CS cell line growth *in vitro* and *in vivo* in a xenograft model ^29, 30^. Despite the promising preclinical reports, a randomized, double-blind, placebo-controlled phase 2 trial of IPI-926 (saridegib) in patients with advanced CS was negative ^31^. Furthermore, a separate single-arm phase 2 trial of the SMO inhibitor vismodegib (GDC-0049) was similarly discouraging, as it did not meet the primary endpoint of 6-month clinical benefit ^32^. Despite these disappointing results, new and more-potent HH pathway inhibitors are in development ^28^.

## Tumor suppressor pathways

Perturbations to tumor suppressor pathways are common in CS. Alterations in the retinoblastoma (Rb) tumor pathway were observed in 33% (whole-exome sequencing) to 96% (immunohistochemistry [IHC]) of cases of conventional and dedifferentiated CS ^27, 33^. Rb binds to and inhibits the E2F transcription factor to restrict G1 to S phase of the cell cycle division. This may occur through loss of heterozygosity (LOH) of Rb in grade 3 CS and dedifferentiated CS ^34^, increased expression of cyclin-dependent kinase 4 (CDK4) or cyclin D1, or reduced expression of CDKN2A/p16 ^33^. There are multiple CDK4/6 inhibitors (palbociclib, ribociclib, and abemaciclib) approved for the treatment of hormone receptor-positive breast cancer in combination with hormone therapy. CDK4/6 inhibitors are a rational approach to test in advanced CS.

*TP53* is the most commonly mutated gene in human tumors. Likewise, alterations in the *TP53* gene occur in 20–50% of conventional and dedifferentiated CS ^27, 35^. p53 protein inactivation may also occur by binding to mouse double minute 2 homolog (MDM2). Overexpression of MDM2 by IHC was reported in 33% of high-grade CS, and MDM2 mRNA expression correlated with increasing histological grade ^33^. These results suggest that strategies to block the p53–MDM2 interaction with small molecule antagonists like RG7112 (F Hoffman-La Roche) to restore p53 function may be a therapeutic avenue to explore in this subset of CS similar to its investigation in well-differentiated liposarcomas ^36^.

## PI3K–Akt–mTOR pathway

Membrane receptor tyrosine kinase (RTK) activation triggers activation of the lipid kinase PI3K, which generates PIP3. The serine-threonine kinase Akt is activated in part by binding of PIP3. Akt regulates multiple biological processes including cell survival, proliferation, growth, and glycogen metabolism. Phosphatase and tensin homolog (PTEN) is a lipid phosphatase that catalyzes the dephosphorylation of PIP3 to negatively regulate Akt. Loss of PTEN function has been implicated in many human cancers; however, *PTEN* mutations are rare in CS despite evidence of active Akt signaling ^37, 38^.

The mammalian target of rapamycin (mTOR) kinase regulates cell cycle progression, cellular proliferation and growth, autophagy, and angiogenesis by integrating energy and nutrient status and PI3K/AKT signaling ^39^. S6 phosphorylation, a surrogate of PI3K/mTOR activation, was observed in 73/106 (69%) of conventional and 11/25 (44%) of dedifferentiated CS clinical samples ^38^. Treatment with BEZ235, a dual PI3K/mTOR inhibitor, inhibited the growth of all CS cell lines tested and G1 arrest without induction of apoptosis ^38^. A combination of the mTOR inhibitor sirolimus and low-dose daily cyclophosphamide in 10 patients with advanced conventional CS resulted in one (10%) objective response, and six (60%) patients had stabilization of disease for at least 6 months ^40^. Investigation of PI3K–Akt–mTOR pathway inhibition in CS should continue.

## microRNA

MicroRNAs (miRNAs) are 18–24 nucleotide length, small noncoding RNAs that regulate eukaryotic gene expression at the post-transcriptional level by binding to 6–8 nucleotides at the 3’ untranslated region (3’ UTR) of their target mRNAs. The short miRNA–mRNA binding site permits each miRNA to target multiple mRNAs. There are nearly 600 miRNAs in the human genome, which target approximately 60% of all human genes ^41, 42^.

miRNAs are involved in normal chondrogenesis. For example, miR-140 negatively regulates histone deacetylase 4 (HDAC4) in non-hypertrophic chondrocytes ^43^. HDAC4 regulates the chondrocyte hypertrophic phase by inhibiting runt-related transcription factor 2 (RUNX2). When miR-140 binds HDAC4, RUNX2 inhibition is removed, and chondrocyte hypertrophy is enhanced prior to entering the apoptotic phase before ossification. Not surprisingly, miRNAs are also intimately involved in CS oncogenesis. miR-100 is a tumor suppressor that targets and inhibits mTOR and is downregulated in CS ^44^. Forced expression of miR-100 sensitizes CS cell lines to cisplatin. Similarly, miR-30a decreases tumor proliferation, migration, and invasion through targeting of oncogenic SRY-related HMG box 4 (SOX4) ^45^. In contrast, miR-181a is considered a CS oncogene, as it is overexpressed in high-grade CS, is upregulated by hypoxia, and increases vascular endothelial growth factor (VEGF) expression by targeting regulator of G-protein signaling 16 (RGS16), a negative regulator of CXC chemokine receptor 4 (CXCR4) signaling ^46^. These studies demonstrate the complex interplay of miRNA in CS oncogenesis.

## Epigenetics

Post-transcriptional gene regulation is caused by DNA methylation and histone modification. In CS, the promoter of the tumor suppressor gene *P16 ^INK4a^* is hypermethylated ^47^. Similarly, hypermethylation of the promoter region of the tumor suppressor *RUNX3* transcription factor leads to reduced gene expression, increased proliferation, and reduced apoptosis in CS cells *in vitro* ^48^. Expression of RUNX3 correlates with improved CS clinical outcomes, further underlying the importance of DNA methylation in CS ^48^. Naturally, the postulated mechanism is mutant *IDH*-induced accumulation of D2HG leading to inhibition of the demethylase TET methylcytosine dioxygenase proteins. However, a recent report suggests alternative mechanism(s) are responsible ^49^. This analysis of 92 central and 45 peripheral CS tumors showed that although the CS were strongly positive for the H3K4me3, H3K9me3, and H3K27me3 histone modifications, neither these modifications nor the variable levels of 5-hydroxymethylcytosine and 5-methylcytosine correlated with *IDH1/2* mutation status.

Inhibition of DNA methyltransferase in the H-EMC-SS CS cell line with decitabine restored expression of the hypermethylated gene encoding HS 3-O-sulfotransferase 2 (HS3ST2), an enzyme essential for HS biosynthesis ^50^. This resulted in reduced proliferation, invasion, and adhesion. However, in the Swarm rat CS (SRC) model, global demethylation induced by decitabine led to CS progression *in vitro* and *in vivo* ^51^. These conflicting results indicate significantly more research is needed before therapeutic targeting of epigenetic modification can proceed.

## Integrins

Integrins are a family of heterodimeric transmembrane adhesion receptors which mediate cell–cell and cell–extracellular matrix (ECM) interactions. To date, 24 distinct integrin heterodimers have been identified, generated from various combinations of 18 α- and 8 β-subunits. Integrin signaling interacts between both growth factor and chemokine signaling. As a result, integrins play critical roles in cancer cell migration, invasion, and metastasis through their two principal functions: attachment of the cell to the ECM and signal transduction from the ECM to the cell ^52^.

CS consists of abundant ECM with few dividing cells and poor vascularity ^53^. This may account for its primary resistance to both chemotherapy and radiation therapy. In CS, growth factors and chemokines/cytokines control the expression of specific integrins to promote cell migration. For example, insulin-like growth factor-1 (IGF-1) increases the migration of CS cells by increasing αvβ1 integrin expression ^54^, and CXCL12/SDF-1 chemokine, which is normally secreted by the lung epithelium, enhances the invasiveness of CS cells by increasing αvβ3 integrin expression through the CXCR4/ERK/NF-κB pathway ^55^. This may account for the tropism for lung metastases. Despite preclinical rationale for targeting integrin signaling, late-phase clinical trials of a synthetic peptide integrin antagonist (cilengitide) with radiotherapy in glioblastoma and an anti-αv-integrin antibody (abituzumab) in combination with chemotherapy and cetuximab (anti-EGFR antibody) in colorectal cancer have reported disappointing results ^56, 57^. These results highlight the complexity of targeting diverse integrin family members with significant redundancy.

## Angiogenesis

Expression of the proangiogenic ligand VEGF is dependent upon hypoxia-inducible factor-1α (HIF-1α), which is upregulated under hypoxic conditions ^58^. Both normal and malignant chondrocytes induce HIF-1α expression during hypoxia, and HIF-1α expression directly correlates with reduced disease-free survival ^59^. Further, CS vascularity increases with increased histologic grade ^60^. Accordingly, our phase 2 study of pazopanib, a multi-targeted receptor tyrosine kinase inhibitor (including VEGF-R) (NCT01330966), in patients with unresectable or metastatic CS has completed accrual and is currently undergoing analysis for safety and efficacy. Pending these results, inhibition of angiogenesis may be another therapeutic avenue for CS.

## Immunotherapy

Immune checkpoint blockade with anti-PD1 (programmed death 1) and anti-PD-L1 (PD-ligand 1) antibodies has transformed solid tumor oncology. Positive clinical outcomes have generally correlated with tumor mutational burden (TMB) and PD-L1 expression ^61^. In contrast, sarcomas generally have quiescent genomes and low TMB; CS were in the lowest decile in an analysis of TMB in over 100,000 cancer genomes in 167 distinct cancer types ^62^. In addition, PD-L1 expression in sarcomas is low ^63^. In a phase 2 trial of the anti-PD-1 antibody pembrolizumab, conducted by the Sarcoma Alliance for Research through Collaboration (SARC), 1 out of 5 (20%) subjects with dedifferentiated CS experienced RECIST response ^63^. However, in a French multi-center, phase 2 trial of pembrolizumab with metronomic cyclophosphamide, 1 out of 50 patients with soft-tissue sarcoma (STS) experienced response, and 0 out of 2 subjects with extraskeletal CS responded ^64^. Additionally, the Alliance for Clinical Trials in Oncology conducted a randomized phase 2 trial of the anti-PD-1 antibody nivolumab alone or in combination with the anti-CTLA4 antibody ipilimumab in patients with STS or bone sarcoma ^65^. The overall response rate was 5% with nivolumab monotherapy and 16% with the combination. However, bone sarcomas accounted for only 12% of the monotherapy cohort and 10% of the combination therapy cohort. Furthermore, only 1 dedifferentiated CS was in each cohort, and neither experienced response. Novel checkpoint blockade combinations will likely be required to advance immunotherapy in CS.

## Summary

Advances in CS genetics, epigenetics, and biology have significantly informed this disease’s underlying oncogenesis. However, this has yet to translate into substantial advancements in therapy. Sustained research will be needed in this rare and sometimes deadly cancer. Novel therapeutic breakthroughs will likely be a result in the not-too-distant future. It is probable that combination therapy targeting multiple pathways will be required to effectively treat advanced CS.
